# Gut Microbiota and Brain Aging: Identifying Keystone Biomarkers for Cognitive Health

**DOI:** 10.3390/biomedicines14091975

**Published:** 2026-09-02

**Authors:** Muskan Bhatia, Sidharth P. Mishra, Raghvendra K. Mishra, Shalini Jain, Hariom Yadav, Rajesh S. Tomar

**Affiliations:** 1 Amity Institute of Biotechnology, Amity University Madhya Pradesh, Gwalior 474005, India; muskanbhatia1981@gmail.com (M.B.); rkmishra@gwa.amity.edu (R.K.M.); 2 USF Center for Microbiome Research, USF Microbiome Institute, Morsani College of Medicine, University of South Florida, Tampa, FL 33620, USA; mishras219@usf.edu (S.P.M.); jains10@usf.edu (S.J.); 3 Center of Excellence for Aging and Brain Repair, Morsani College of Medicine, University of South Florida, Tampa, FL 33620, USA; 4 Department of Neurosurgery, Brain and Spine, Morsani College of Medicine, University of South Florida, Tampa, FL 33620, USA

**Keywords:** blood–brain barrier (BBB), aging biomarkers, neurodegenerative disorders, precision medicine, gut–brain axis

## Abstract

The fact that the population is getting older has greatly increased the occurrence of cognitive decline and neurodegenerative diseases, underlining the importance of having reliable biomarkers that can measure biological aging before irreversible neurological damage takes place. New evidence shows that brain aging is not just the result of changes within neurons themselves but is also greatly affected by the gut microbiota via immune, metabolic, endocrine, and neurovascular signaling. This review brings together the existing knowledge about biomarkers of biological aging-such as telomere shortening, epigenetic clocks, oxidative stress, inflammation, cellular senescence, and metabolic dysfunction-as well as established cognitive biomarkers obtained from neuroimaging, cerebrospinal fluid, blood, genetic evaluations, and neuropsychological tests. We also point out that changes associated with age in the composition of the gut microbiota and the metabolites it produces are becoming more and more involved in the mechanisms connecting intestinal dysbiosis, dysfunction of the blood-brain barrier (BBB), neuroinflammation, and age-related cognitive decline. Through this approach of combined and complementary biomarker systems, we hypothesize that the gut microbiota has emerged as a central regulator of biological and cognitive aging and may provide a useful source for development of biomarkers of cognitive resilience and risk of neurodegenerative diseases. Lastly, we consider microbiome-based interventions, including probiotics, prebiotics, dietary changes, fecal microbial transplant, and new treatment modalities derived from molecular studies, as possible approaches to the prevention and management of age-related cognitive decline. Collectively, this review provides a comprehensive framework linking aging biology, microbiome science, and cognitive biomarkers to advance biomarker-driven precision medicine for healthy brain aging.

## 1. Introduction

Population aging has emerged as one of the greatest global healthcare challenges of the twenty-first century, accompanied by a rapid increase in age-associated disorders, particularly cognitive impairment and neurodegenerative diseases such as Alzheimer’s disease (AD). Aging is characterized by a progressive decline in physiological function driven by interconnected molecular mechanisms, including cellular senescence, epigenetic alterations, oxidative stress, chronic low-grade inflammation [inflammaging], mitochondrial dysfunction, and metabolic dysregulation [1,2]. These biological alterations collectively accelerate functional deterioration across multiple organs, including the brain, thereby increasing susceptibility to cognitive decline and dementia.

There is recent evidence to indicate that the gut microbiota plays an important role in healthy aging as a result of its wide-ranging interactions with host metabolism, immune function, the integrity of the intestinal barrier, and neuroendocrine signaling. Instead of operating independently, the biology of aging and the gut microbiome are linked together via the microbiota-gut-brain axis, a bidirectional communication system that is mediated by microbial metabolites, immune signaling, endocrine pathways, and neural circuits [3]. As a result, changes in the composition and function of gut microorganisms have been connected to biological aging and cognitive resilience. Both our own recent studies and those of other researchers have underlined the crucial role of gut microbial dysbiosis, intestinal barrier dysfunction, and chronic inflammation in metabolic diseases, stressing the microbiome as a common mechanistic link between systemic aging and age-related disorders [4].

Among the many microbial and molecular signatures that are associated with gut–brain interactions, some biomarkers might have particularly strong impacts on disease-related pathways, making them potential ‘keystone’ indicators [5]. Neuroimaging biomarkers, such as hippocampal atrophy, white matter hyperintensities, amyloid-β [Aβ] deposition, and tau pathology, offer important insights into the process of neurodegeneration. Cerebrospinal fluid biomarkers, including Aβ42, phosphorylated tau, and neurofilament light chain, along with newly emerging blood-based biomarkers, allow for increasingly sensitive detection and monitoring of cognitive decline [6]. Furthermore, genetic risk factors such as APOE ε4 and TREM2 variants, as well as synaptic proteins like synaptophysin and SNAP25, also contribute to better risk stratification and disease prediction [7,8]. Apart from these neurological biomarkers, systemic aging biomarkers, including telomere attrition, epigenetic clocks, p16INK4a expression, the senescence-associated secretory phenotype (SASP), inflammatory mediators (CRP, IL-6, and TNF-α), and metabolic regulators such as insulin-like growth factor-1 (IGF-1) and the mammalian target of rapamycin (mTOR), reflect the biological processes that contribute to both aging and neurodegeneration [9,10,11]. At the same time, the gut microbiome experiences significant changes associated with age, marked by a decrease in microbial diversity, a modification of its microbial composition, and functional metabolic changes [12]. These changes affect the production of microbial metabolites such as short-chain fatty acids (SCFAs), bile acids, trimethylamine N-oxide (TMAO), ethanolamine, spermidine, and taurine, all of which play a role in maintaining intestinal barrier integrity, immune homeostasis, mitochondrial function, and neuronal signaling [13]. Dysbiosis has been linked to immunosenescence, systemic inflammation, increased intestinal permeability, and blood-brain barrier dysfunction, factors that may lead to neuroinflammation and cognitive decline [14,15,16,17]. Furthermore, experimental studies have shown that microbial metabolites can regulate hippocampal neurogenesis, synaptic plasticity, neurotransmitter synthesis, and hypothalamic-pituitary-adrenal (HPA) axis activity, thus highlighting the gut microbiota as an active participant in brain aging rather than simply acting as a passive observer [18]. These ideas are in close agreement with our latest reviews, which emphasize microbiota-mediated barrier dysfunction and microbial metabolites as major regulators of chronic inflammatory diseases [19].

There is increasing evidence to show that biological aging, cognitive decline, and gut microbial dysbiosis all involve the same mechanisms, such as inflammaging, metabolic dysfunction, mitochondrial impairment, oxidative stress, insulin resistance, and immune dysregulation [20,21,22]. Because of these common pathways, our understanding of the current paradigm has changed from seeing the gut microbiota as merely an environmental modifier to considering it as an essential part of precision medicine. As a result, various microbiome-targeted interventions, such as probiotics, prebiotics, postbiotics, dietary interventions, fecal microbiota transplantation, microbial metabolites, and personalized microbiome-guided therapeutics, are now being studied as possible ways of maintaining cognitive resilience and delaying the progression of neurodegenerative diseases [23,24,25,26,27,28].

The evidence relating the gut microbiome to aging and cognitive decline comes from a variety of sources, including mechanistic studies, experimental models, observational studies in humans, and clinical investigations, which is why the strength of this evidence differs according to the particular aspect of the microbiota-gut-brain axis. In this review, the findings are understood in the context of this hierarchy of evidence so as to differentiate well-established clinical observations from newer mechanistic insights and preclinical evidence.

Even though earlier reviews have each separately summarized biomarkers of aging, of cognitive impairment, or of the gut microbiome, these biomarker systems are usually assessed on their own. Biological aging, cognitive decline, and gut microbial dysbiosis all come about through interconnected mechanisms, which include microbial metabolism, intestinal barrier dysfunction, chronic inflammation, immune dysregulation, and neurovascular signaling. In order to fill this gap, we are proposing an evidence-based hierarchical framework that brings together three complementary biomarker areas: systemic biomarkers of biological aging, established neurological biomarkers of cognitive impairment, and microbiome-derived biomarkers that have a mechanistic link between intestinal and brain health. A keystone biomarker should ideally show mechanistic relevance, have reproducible associations in separate studies, possess predictive ability, allow for reliable quantification, and have potential clinical usefulness. Rather than just looking at the biomarkers, this review critically looks at how these interconnected biomarker systems supplement one another, assesses the strength of the current evidence, and discusses their translational potential for the early diagnosis of disease, for risk stratification, for monitoring the course of the disease, and for microbiome-targeted therapeutic strategies aimed at achieving healthy brain aging.

The studies referred to in this review were found by searching PubMed, Web of Science, and Scopus for research relating to the gut microbiota, biological aging, cognitive decline, and the gut-brain axis. The search mainly concentrated on articles published between 2000 and 2026, although earlier landmark papers were also included if they offered important background information or had established key concepts in the field. The search used combinations of the following terms: gut microbiota, gut microbiome, brain aging, cognitive decline, Alzheimer’s disease, gut-brain axis, aging biomarkers, microbiota-derived metabolites, short-chain fatty acids, trimethylamine N-oxide, intestinal barrier, neuroinflammation, and healthy aging. We included in this review peer-reviewed original research articles, systematic reviews, meta-analyses, and clinical studies that had been published in English. Whenever possible, we gave priority to evidence from human studies, while also including well-designed animal and mechanistic studies in order to gain biological insight into the interactions between the gut microbiota and the host as well as into the mechanisms of gut-brain communication. The studies cited were chosen on the basis of their relevance to the aims of this review, their scientific quality, their contribution to understanding the mechanisms involved, and their potential for translational significance.

## 2. Biology of Aging and Its Biomarkers

Aging is a slow, irreversible physiological process. It is characterized by reduced tissue and cellular function, along with substantially increased risk of developing various aging-related diseases, including neurodegenerative disorders, cardiovascular disease (CVD), metabolic disorders, musculoskeletal problems, and immune system diseases [2]. There are many biomarkers of aging, which refer to physiological and molecular signs that reflect age-related declines in structure or function across the body, organs, tissues, cells, and subcellular components [29,30]. There are many biomarkers that are directly connected to aging and which can be of value in detecting various processes that occur or play a major role during the aging process, for instance, markers of cellular senescence, such as telomere length, p16INK4a, and SASP; markers of oxidative stress, such as the levels of reactive oxygen species (ROS) and antioxidant enzymes (for example, superoxide dismutase); inflammatory markers, such as CRP, IL6, TNFα, and other cytokines linked to inflammaging; and metabolic biomarkers, including IGF1, mTOR signaling, and mitochondrial function [31]. Firstly, markers of cellular senescence include p16INK4a, which is a regulator of the cell cycle and a key biomarker of cellular aging; it also functions as a tumor suppressor by inhibiting cyclin-dependent kinases 4 and 6 [30,32]. In certain tissues, such as adipose tissue, skeletal muscle, and the eye, where p16Ink4a is involved in the development of age-related diseases, continuously getting rid of cells that express p16Ink4a delays the onset of these characteristics [33,34]. Furthermore, when these senescent cells are removed later in life, the progression of pre-existing age-related conditions is slowed (Table 1).

Aging is a multifactorial physiological process that occurs throughout life, characterized by structural and functional changes at different molecular levels that lead to gradual tissue degeneration. Physiological aging involves progressive functional decline, leading to the loss of homeostatic control, which predisposes the organism to age-related pathologies, including neurocognitive decline, cardiovascular diseases, metabolic dysfunction, musculoskeletal deterioration, and immunological disorders. Cellular senescence is a permanent cell-cycle arrest state that is provoked by various stressful stimuli, including telomere shortening and DNA damage, oxidative stress, oncogenic signaling, and others. While reversible in young organisms, senescence becomes irreversible with age. Senescent cells accumulate in tissues during aging and can be recognized by the senescence-associated secretory phenotype (SASP). The SASP plays an essential role in aging by driving age-associated pathologies such as neurodegeneration, cancer, osteoarthritis, cardiomyopathies, atherosclerosis, and chronic inflammatory disorders [45]. Exosomal SASP (eSASP) differs from soluble SASP (sSASP). Therefore, eSASP can be used as a diagnostic biomarker [45]. Aging is a continuous and complex biological process influenced by genetic, environmental, random, and endogenous factors. Epigenetic clocks are one of the new and robust biomarkers of aging that can predict human healthspan and mortality [49]. They are based on DNA methylation (DNAm) and represent the most important and objective measure of chronological age [49]. DNAm clocks were initially based on CpG sites, the sites where a cytosine is followed by a guanine, with variations in methylation levels reflecting changes in gene expression and cellular function. Epigenetic clocks can predict biological aging and healthspan with different approaches. There are several DNAm clocks, including Horvath’s clock, which is based on 353 CpG sites and represents a first-generation epigenetic clock [50]; Hannum’s clock, based on 71 CpG sites [50] and mostly associated with hematopoietic tissues, developed to study blood aging; and PhenoAge or Levine’s clock, based on 513 CpG sites [51]. These clocks are strongly associated with immune aging and inflammatory processes.

Another set of biomarkers is oxidative stress markers, which are theoretically important for aging but are not yet measured in a way that allows large-scale collection. Elevated levels of ROS, which are crucial for cellular signaling, can cause considerable damage to cellular structures [52]. It has been proposed that ROS contribute significantly to the development of age-related muscle mass decline [sarcopenia], alterations in the CNS, hearing impairment, and Parkinson’s disease and AD [53,54]. Meanwhile, intrinsic antioxidants such as superoxide dismutase (SOD) and glutathione peroxidase, along with extrinsic antioxidants such as vitamins A, B, C, and E, mitigate aging and related ailments by counteracting oxidative stress [55]. Research indicates that SOD might act as a tumor suppressor, while carotenoids could offer protective benefits against both CVD and cancer [46].

Inflammation markers are another category of markers. These are often elevated in older adults and linked to age-related chronic diseases and disability and are, therefore, referred to as “inflammaging markers”. Inflammatory markers such as CRP are response proteins produced in the liver and indicate the levels of inflammation [56]. CRP levels rise as part of the immune response to infection and tissue injury and may be elevated in chronic conditions such as T2D, asthma, rheumatoid arthritis, and heart disease [56]. IL6 is a key inflammatory cytokine and constantly increases with age, thus leading to low-grade chronic inflammation, cognitive decline, and mortality [57]. Another inflammatory biomarker, TNFα, also plays an important role in the aging process. TNF*α* is linked to inflammation and increases in aged individuals compared with young controls [58]. Another set of biomarkers is metabolic biomarkers. It includes free IGF1, a growth factor that regulates cell growth and development and inhibits programmed cell death, thereby promoting the progression of diseases like cancer [59,60]. mTOR signaling regulates the speed at which cells and tissues undergo aging, and inhibition of mTOR has shown promise in experimental models to enhance lifespan; meanwhile, mitochondrial function significantly declines with age [61]. The accumulation of somatic mitochondrial tDNA mutations with age may contribute to a decline in mitochondrial function. Therefore, epigenetic clocks serve as an effective means of assessing and understanding biological aging, providing valuable insights into age-associated diseases, lifespan, and potential treatments. As research progresses, they show potential for personalized medicine, helping customize lifestyle choices and therapeutic strategies to enhance both health and lifespan.

## 3. Aging-Related Cognitive Impairment and Its Biomarkers

Age-related cognitive decline is marked by gradual loss of various cognitive abilities such as memory, attention, executive function, and processing speed, linked to neurodegenerative conditions like Alzheimer’s disease (AD) and other forms of dementia [35]. Discovering accurate biomarkers is essential for diagnosis, prognosis, disease progression, and treatment options. Current biomarkers can be categorized into neuroimaging, cerebrospinal fluid (CSF) biomarkers, blood biomarkers, genetic biomarkers, synaptic dysfunction biomarkers, and cognitive biomarkers, indicating different pathologies.

**Neuroimaging biomarkers:** Neuroimaging biomarkers are the main markers used in detecting and monitoring neurodegenerative diseases, especially AD, using computed tomography (CT), magnetic resonance imaging (MRI), and positron emission tomography (PET) [62]. Biomarkers like brain atrophy, which includes a reduction in hippocampal size, are examples of gradual reductions in brain volume caused by aging because of the loss of connections in cells and deterioration of tissues, leading to cognitive impairment. When this occurs, it leads to the development of neurodegenerative diseases like AD [63]. White matter hyperintensities (WMHs) are other changes in the brain that occur due to aging and cognitive impairment. The Study of Health in Pomerania assessed the relationship between WMH burden and brain atrophy associated with aging and AD. Using automated segmentation and machine learning approaches, the study found that people with high WMH burden had low SPARE-BA and high SPARE-AD indices, implying faster brain aging and an AD atrophy pattern [64].

**Amyloid and tau pathology biomarkers:** Cognitive decline with aging results from the presence of proteins in the brain; among them, Aβ plaques are pathological proteins visualized through neuroimaging technologies [65]. Amyloid-β plaque deposition, which occurs due to altered neuronal connections, leads to memory impairments and other cognitive deficits that increase with aging. It is noteworthy that another abnormal protein related to Alzheimer’s disease is tau protein that forms neurofibrillary tangles. Neurofibrillary tangles are abnormal aggregates of filamentous tau proteins linked to pathological changes in the brain, particularly in patients diagnosed with AD [66]. Aβ42 and phosphorylated tau proteins are two other biomarkers that can be detected in the cerebrospinal fluid (CSF) as well as neuroimaging biomarkers indicating aging-associated cognitive decline due to brain pathology. CSF biomarkers, in particular Aβ and tau proteins, play a critical role in the diagnosis of cognitive impairment and AD [36]. Aβ peptides result from the cleavage of amyloid precursor protein (APP) that is predominantly present in neurons as a transmembrane protein. Two pathways, namely, the amyloidogenic and non-amyloidogenic pathways, mediate the metabolism of APP [37]. The former pathway results in the generation of Aβ from APP via BACE1 and γ-secretase-mediated reactions [67]. Consequently, Aβ peptides accumulate, forming plaques, one of the pathological hallmarks of AD. In contrast, the non-amyloidogenic pathway involves α-secretase-mediated APP cleavage that leads to the production of a neurotrophic peptide, APP, and the prevention of Aβ generation [68]. Hence, the balance between these two pathways should be maintained to avoid excessive Aβ production that contributes to the development of AD.

Inflammatory and barrier-related biomarkers: AD is an age-related disease characterized by impaired blood–brain barrier (BBB) and gastrointestinal tract integrity, resulting in enhanced permeability that allows polysaccharides, microbes, and amyloids to enter the central nervous system (CNS) and trigger inflammation [38].

Tau phosphorylation biomarkers: Besides Aβ, another abnormal protein associated with cognitive decline and AD is phospho-tau. CSF phospho-tau biomarkers contribute to the diagnosis of age-related cognitive impairments and AD. It is reported that in phospho-tau proteins, serine and threonine amino acids are subjected to post-translational modifications, leading to the addition of phosphate groups [69]. In patients diagnosed with AD, tau proteins are abnormally phosphorylated, forming neurofibrillary tangles linked to neuronal damage, cognitive decline, and memory impairments. It is noteworthy that the abnormal phosphorylation of tau proteins is observed during normal aging and AD progression. Several studies have revealed the diagnostic value of p-tau181, p-tau217, and p-tau231 in predicting cognitive decline associated with AD [70].

**Neurodegeneration biomarkers:** One component of neurofilaments (NFPs) is the neurofilament light chain (NfL). This protein functions as a biomarker for the diagnosis and prognosis of neurodegenerative disorders. Research on the role of NfL and its connection to cognitive decline in elderly individuals is not widespread; nevertheless, increased concentrations of NfL in blood and CSF have been shown to be related to MCI, AD, and ALS [43]. Considering that there is a significant correlation between NfL in the plasma and CSF, plasma NfL is regarded as an easily accessible and cost-effective biomarker.

**Blood biomarkers**: Blood biomarkers seem to have good potential for non-invasive and relatively cheap detection of age-related cognitive impairment and the development of neurodegenerative diseases compared with CSF and neuroimaging biomarkers. Specifically, among blood biomarkers, plasma Aβ, tau, and neuroinflammatory markers play an essential role in the determination of pathological changes related to cognitive decline. For instance, the plasma Aβ42/Aβ40 ratio is considered the most prominent blood biomarker indicating the presence of amygdala plaques, since a decrease in this ratio is connected to an increase in the amount of brain amyloid deposits, leading to a higher risk of cognitive impairment [71]. Meanwhile, tau proteins (p-tau181, p-tau217, and p-tau231) are considered to reflect pathological changes related to tau accumulation and are associated with the level of cognitive dysfunction. The diagnostic accuracy of plasma tau biomarkers is comparable to that of CSF p-tau and tau PET, while being much more accessible in terms of invasion and cost [72].

Neuroinflammatory biomarkers (interleukin-6 (IL-6), tumor necrosis factor-α (TNFα), and C-reactive protein (CRP)) indicate systemic inflammation that has been associated with neurodegeneration and vascular cognitive impairment [73].

**Genetic biomarkers:** Genetic biomarkers are genetic sequences that can help in determining disease susceptibility, as well as diagnosing and treating the condition [74]. Genetic biomarkers linked to AD include apolipoprotein E ε4 (APOE ε4), a gene variant found to be involved in accelerated cognitive decline and Aβ clearance dysfunction, which are typical pathological mechanisms of AD. TREM2, an activator of innate immunity, is another genetic marker of AD, where a deficiency of which leads to a dysfunctional immune response and higher dementia risk [75]. In addition, there are numerous genetic predispositions to developing AD, including amyloid precursor protein (APP) and mutations in presenilin 1 (PSEN1) and presenilin 2 (PSEN2), which are linked to the development of familial early-onset AD [76]. Furthermore, variations in methylenetetrahydrofolate reductase (MTHFR), peroxisome proliferator-activated receptor gamma (PPARG), glucokinase regulatory protein (GCKR) and ADAMTS family genes are implicated in insulin resistance and metabolic processes [77].

**Synaptic dysfunction biomarkers:** Biomarkers of synaptic dysfunction indicate synaptic degeneration; one of the critical protein changes is the reduction in the synaptic protein synaptophysin, which leads to decreased synaptic density and is associated with impaired cognitive function. In addition, SNAP25 and growth-associated protein 43 (GAP43) in CSF are sensitive to early synaptic damage, serving as synaptic damage indicators [78].

**Cognitive assessments:** Besides biological biomarkers, cognitive tests are also an essential instrument for determining age-related cognitive decline and evaluating mental functions such as memory, language, thinking skills, and attention in the elderly population [79]. These tests help determine the first manifestations of dementia and neurodegenerative disorders and take appropriate measures to manage them. The Mini-Mental State Examination (MMSE) is one of the most used tests for dementia and evaluates the level of cognitive functioning from 0 to 30; the lower the score, the higher the level of dysfunction [80]. The Montreal Cognitive Assessment (MoCA) is known to be more sensitive to the detection of MCI and AD than MMSE [47]. There are other tests, like the abbreviated mental test score (AMTS), Alzheimer’s Disease Assessment Scale-Cognitive (ADAS-Cog), and General Practitioner Assessment of Cognition (GPCOG) [81,82,83].

The application of all these biomarkers, from amyloid to tau, neurodegeneration, synaptic loss, inflammation, genetics, and cognition, is the general approach that may allow us to identify cognitive declines, recognize risk factors, track the progress of the disease, and create treatment methods related to the aging process.

## 4. Microbiome and Aging

The gut microbiome includes a diverse group of microbes such as bacteria, archaea, fungi, viruses, and more, which play important roles in nutrition, metabolism, immune function, intestinal integrity maintenance, and two-way signaling along the gut–brain axis. The process of gut dysbiosis is no longer viewed as an unbalanced relationship between “good” and “bad” microbes but rather as an imbalance in microbial population structure, function, and interactions between host and microbes that disrupts physiological balance [84]. Crucially, the compositions of the microbial communities differ from one person to another due to genetic, nutritional, behavioral, therapeutic, geographic, co-morbidity, and environmental factors. It is important to consider the context of everyone while discussing age-related changes in the microbiome.

**Figure 1:**
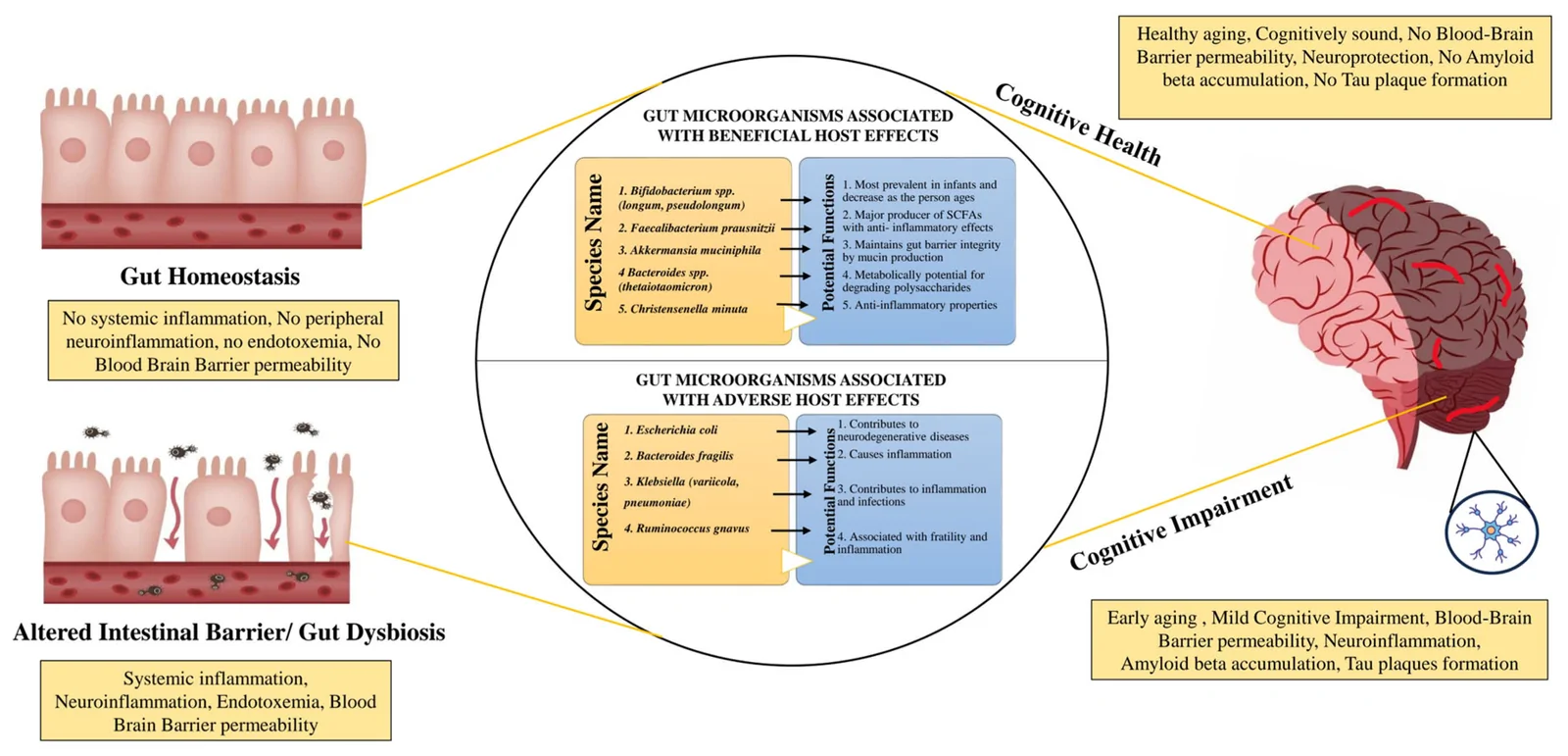
Influence of keystone gut microorganisms on brain health via the gut–brain axis. This diagram compares the effects of beneficial versus pathogenic gut microorganisms on cognitive outcomes. A healthy gut, supported by keystone species such as *Bifidobacterium* spp., *Faecalibacterium prausnitzii*, *Akkermansia muciniphila*, *Bacteroides* spp., and *Christensenella minuta*, is associated with maintenance of epithelial integrity, neuroprotective effects, and a lower inflammatory response, resulting in healthy aging and a cognitively sound brain. In contrast, a leaky gut dominated by pathogenic species, including *Escherichia coli*, *Bacteroides fragilis*, *Klebsiella* spp., and *Ruminococcus gnavus*, has been associated with systemic inflammation, endotoxemia, and increased blood-brain barrier permeability, potentially contributing to neuroinflammation, amyloid-beta accumulation, Tau pathology, and cognitive impairment. The central circular panel showcases the microbial functions, while peripheral panels depict gut integrity and brain outcomes.

The aging process is characterized by a series of modifications to gut microbiome diversity and functioning that influence immune response, metabolism, and tissue physiology. Older people are more prone to inflammatory pathologies and demonstrate compromised immune system performance in comparison with younger subjects [85]. These aging-associated processes result in chronic low-grade inflammation (“inflammaging”), metabolic disorders, and predisposition to age-related pathologies, such as neurodegeneration [86]. However, not only is aging associated with variations in the microbiome, but diet, exercise, frailty, polypharmacy, pathologies, and institutionalization also significantly impact microbial content in older patients. These factors may alter the relative abundance and functional activity of gut microorganisms, whose effects can vary according to the host and environmental context (Figure 1).

Thus, controlled longitudinal studies applying omics technologies are required to differentiate aging-associated modifications to the microbiome from other factors [87]. Several observations have reported differences in phyla dominance due to aging, including the relative abundance of Firmicutes and Bacteroidetes [88]. It seems that differences in the functional composition of the microbiota can provide more valuable information than the composition of bacterial taxa at the phylum level. During aging, a decrease in the abundance of SCFA-producing bacteria such as Faecalibacterium, Roseburia, and Eubacterium is observed, as well as an increase in the abundance of pro-inflammatory pathobionts.

The metabolic products of the microbiota, especially SCFAs and trimethylamine N-oxide (TMAO), are recognized as modulators of the interaction between the microbiome and the cardiovascular system, aging, and host metabolism [89]. TMAO is formed from dietary choline and L-carnitine under the influence of microbiota and hepatic enzymes and regulates inflammatory response and lipid metabolism, while SCFAs affect the metabolic pathways of lipid breakdown, adipocyte differentiation, and energy homeostasis. [90]. Specifically, acetate and butyrate inhibit lipolysis of fat deposits in the body, while propionate stimulates extracellular lipolysis via induction of lipoprotein lipase expression and lowers blood lipid concentrations, body weight, hypertension, and the development of atherosclerosis [91].

This study provides evidence that SCFAs are key endogenous regulators of intestinal and systemic homeostasis through their multiple effects on the enhancement of the intestinal barrier function, the control of inflammatory responses, and the modulation of the gut-brain axis [41,92]. The decrease in SCFA production is strongly associated with aging processes, frailty, and cognitive dysfunction [42,93]. Moreover, SCFAs were reported to stimulate mucin production and goblet cell expansion as well as to improve glucose tolerance, insulin sensitivity and intestinal barrier integrity, thereby preventing metabolic disorders. Experimental studies suggest that changes in the composition of the gut microbiota that occur with age and are associated with decreased SCFA production may play a role in cognitive decline [94].

The role of TMAO as a promising biomarker for cognitive dysfunction in connection with brain aging and neurocognitive disorders (NCDs) is being actively studied [95]. The high levels of TMAO in the blood are commonly associated with Alzheimer’s disease and cognitive dysfunction in elderly patients [95]. Higher levels of TMAO have been associated with increased neuroinflammation, oxidative stress, and poor synaptic plasticity, and thus contribute to the increased vulnerability of the brain to cognitive dysfunction due to aging. Nevertheless, TMAO blood levels are affected not only by the composition of the microbiota but also by diet, renal and liver function, and medications. Other metabolites that belong to the microbiome group, spermidine and taurine, have also received much attention owing to their potential influence on healthy aging. Experimental studies suggest that spermidine and metformin may reduce aging-associated intestinal permeability and inflammation by increasing the number of goblet cells, enhancing mucus secretion, and inhibiting Wnt signaling [96]. Spermidine is one of the naturally occurring compounds that belongs to the polyamine family. Spermidine is an antioxidant and anti-inflammatory compound that can increase metabolism, regulate protein expression, and enhance autophagy [97]. Spermidine is also associated with increased life expectancy, while low endogenous spermidine levels are linked to age-related disorders [98]. Taurine has the potential to modulate several aspects of physiological aging, like cell senescence, decreased telomerase expression, DNA damage response, and mitochondrial dysfunction. Low taurine levels were connected to various age-related diseases, while physical activity could raise taurine and its metabolite levels [48]. The alterations in the functional activity of the microbiome that occur with age may contribute to decreased intestinal barrier function and increased intestinal permeability, resulting in increased lipopolysaccharide in the circulation and, consequently, endotoxemia and a systemic inflammatory state [99].

Aging-associated gut dysbiosis not only affects immune responses but also increases susceptibility to infection, which has been associated with reduced vaccine responsiveness. Together with the other aging-related changes in cells and molecules, these mechanisms lead to the age-related decline in immune system functions called immunosenescence [100]. Endotoxemia and immunosenescence are characterized by increased levels of IL-1β, IL-6, TNF-α, CRP, and other inflammatory cytokines, which are implicated in neurodegeneration and cognitive dysfunction [101]. Furthermore, this study revealed a bidirectional relationship between the gut microbiota and cell senescence since metabolites derived from bacteria and modified dietary components via microbiota can modulate the cell senescence program in the gut epithelium, while senescent epithelial and stromal cells can affect the microbiota via SASP [19,102]. Therefore, understanding microbiota–cell senescence communication could help discover potential strategies to target both factors and promote healthy aging [103].

The role of microbial function in the aging process is additionally being emphasized by research on healthy longevity. Indeed, long-lived families and centenarians often present distinctive compositions of their gut microbiome contributing to their remarkable longevity [104]. While core microbiomes tend to be relatively stable across different age groups in long-living populations, the microbiome of centenarians appears to be characterized by greater diversity and an enrichment of short-chain fatty acids, secondary bile acids, and amino acids [105]. Some bacterial taxa were linked to longevity, presumably due to their anti-inflammatory properties and the production of essential amino acids [105]. At the same time, Bifidobacterium pseudocatenulatum, Klebsiella, and Ruminococcus are positively associated with brain function and the modulation of essential amino acids [106,107,108]. Nevertheless, the relationship between the microbiome and aging is complex and depends to a considerable extent on the population studied, as well as diet, genetics, and the environment. Therefore, when talking about specific bacteria associated with health status or aging, it is important to consider them in the context of an entire ecosystem and the metabolites that can be produced by it.

It has been reported that the gut microbiota changes qualitatively with age. For instance, people aged 50–60 tend to have higher amounts of *Christensenella minuta* and *Akkermansia muciniphila*, while those aged 60–70 have elevated numbers of Bifidobacterium longum and *Faecalibacterium prausnitzii* [109,110]. Prevotella capri is consistently found in the guts of people aged 70–90 years. Meanwhile, the gut microbiota of people aged over 100 years is characterized by an increased abundance of *Synergistes*, *Escherichia*, *Bacteroides fragilis*, *Ruminococcus* and *Prevotella stercorea* [111], and *Klebsiella* [112] (Table 2). These findings suggest that there is a potential core microbiota that possibly contributes to healthy aging and cognitive function.

### Microbiome-Derived and Beneficial Metabolites for Healthy Aging and Cognitive Improvement

**Figure 2:**
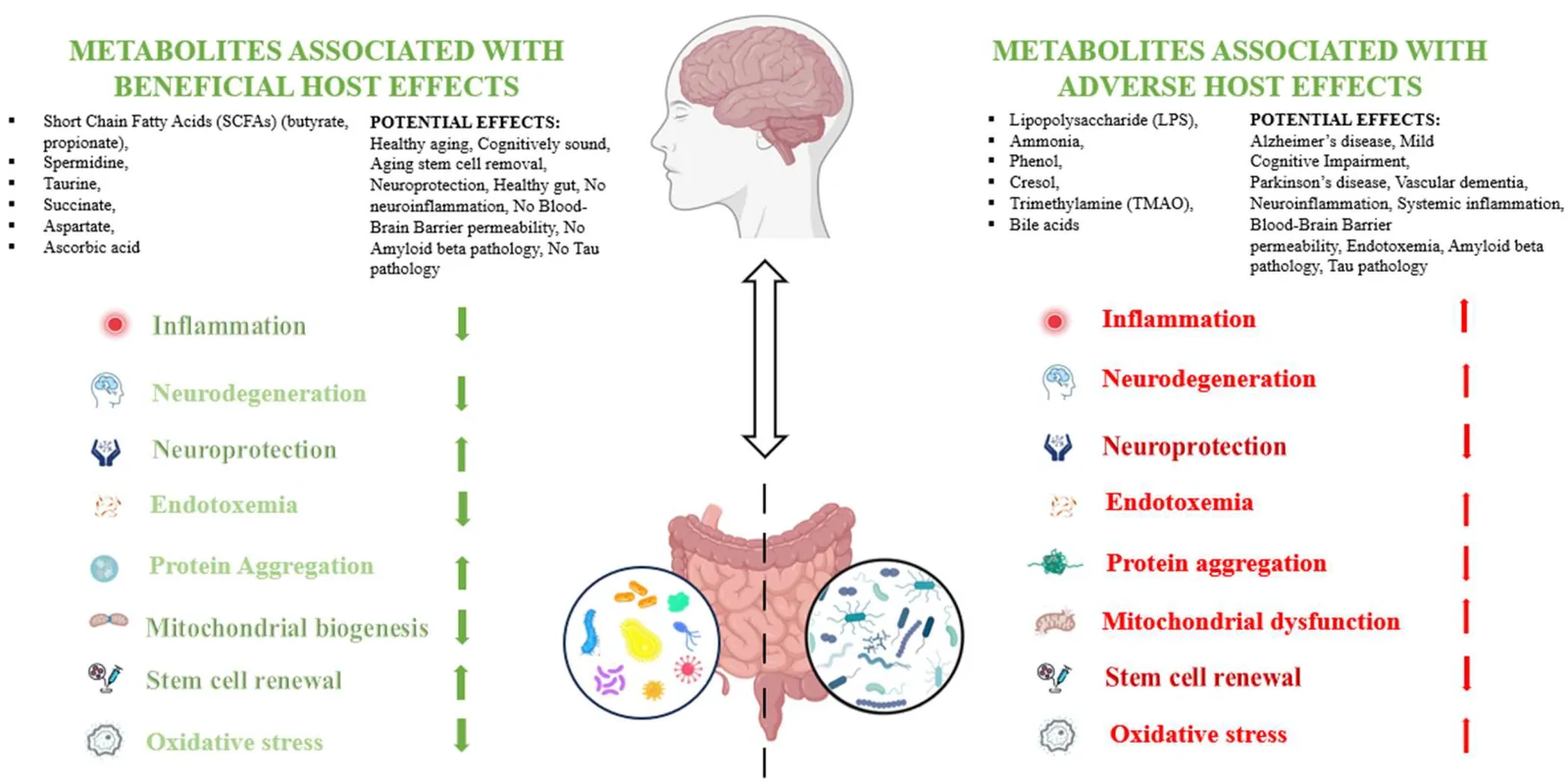
Impact of gut-derived metabolites on brain health via the gut-brain axis. This comparative schematic illustrates how beneficial versus pathogenic gut metabolites influence neurological outcomes. On the left, neuroprotective compounds, including short-chain fatty acids [butyrate and propionate], spermidine, taurine, succinate, aspartate, and ascorbic acid, support healthy aging, reduce inflammation, and maintain blood–brain barrier integrity, preventing neurodegeneration and protein aggregation. On the right, neurotoxic metabolites such as lipopolysaccharide [LPS], ammonia, phenol, cresol, trimethylamine [TMAO], and bile acids promote systemic and neuroinflammation, increase blood-brain barrier permeability, and contribute to pathologies including Alzheimer’s disease, Parkinson’s disease, and vascular dementia. Arrows indicate the direction and magnitude of effects on inflammation, neuroprotection, mitochondrial function, and oxidative stress.

Multiple studies have demonstrated associations among altered gut microbiota, inflammation, and neurodegenerative markers such as Aβ, suggesting that maintaining a balanced microbiome could help preserve health and prevent age-related diseases [117]. These findings underscore the potential of the gut microbiota and its derived metabolites to promote longevity through immune modulation, neuroprotection, and metabolic support [105]. These findings provide a rationale for establishing a new homeostasis of gut microbiota-derived and/or beneficial metabolites (as shown in Figure 2), thereby improving neural and immune function and extending lifespan and cognitive function to achieve healthy aging.

***Short-chain fatty acids (SCFAs)***: Butyrate, acetate, and propionate, the production of which decreases with age, are able to improve gut barrier functions [118]. This, in turn, reduces the translocation of bacteria and the associated inflammation in the gut, which decreases neuroinflammation and improves brain functionality and cognition [119]. Increasing the concentration of SCFAs supports the mucus layer integrity and the gut–brain axis’s functionality and is linked to healthy brain aging [120]. Restoring SCFA levels through dietary or microbiota-targeted interventions enhances mucin expression, supports the integrity of the gut–brain axis, and is associated with healthy brain aging [121].

***Trimethylamine N-oxide (TMAO):*** The production of this metabolite is elevated with age and impacts the whole body, causing age-associated diseases through body-wide inflammation [122]. The increase in TMAO is linked to worse cognitive performance and higher neuroinflammation that can lead to neurodegeneration [123]. Additionally, high TMAO levels are associated with cardiovascular diseases and atherosclerosis development [124]. Following a heart-healthy diet that lowers TMAO levels could reduce the risk of several age-associated diseases [125]. Among others, bile acids are also positively linked to healthy aging. Recent studies have identified an association between the presence of certain bacterial species encoding enzymes that participate in alternative bile acid biosynthesis pathways and extreme longevity [126]. These findings suggest that modulation of the bile acids’ metabolism could be instrumental in designing precision interventions for age-related conditions, including neuroinflammation and cognitive decline.

***Ethanolamine:*** Microbial ethanolamine catabolism has been identified as a possible mechanism for host–microorganism communication and can be seen as a viable biomarker and therapeutic target [127]. Its availability is tightly controlled by microbial ethanolamine utilization (Eut) pathways, which become dysregulated during aging and metabolic disorders, leading to impaired gut barrier function, chronic inflammation, and microbial dysbiosis [19,128]. These alterations contribute to age-related diseases, including obesity, type 2 diabetes, AD, and cognitive decline through systemic inflammation and microbiota–gut–brain axis dysfunction [4,30,129,130]. Thus, microbial ethanolamine metabolism represents a promising keystone biomarker and therapeutic target for precision microbiome-based interventions to promote healthy brain aging [19,131].

***Spermidine:*** With age, spermidine levels decrease, which is linked to impaired cellular functions and reduced autophagy, which, in turn, is associated with the development of several age-associated diseases [98]. Supplementation with exogenous spermidine promotes autophagy and has neuroprotective effects, improving memory and cognitive performance [132], and could be beneficial for brain aging. In particular, spermidine supplementation was shown to improve memory in older people with subjective cognitive decline [39]. Autophagy induction and mitochondrial function support are considered two major mechanisms by which spermidine supplementation could be used to combat age-associated diseases, including AD [98].

***Taurine:*** With age, taurine levels decrease, which is associated with a wide range of age-related diseases [48]. Taurine supplementation prevents memory decline and cognitive impairment in aged mice [133]. Taurine supports mitochondrial function and enhances the production of ATP, thus promoting cell health and longevity [42]. It also participates in the regulation of gut microbiota composition and intestinal barrier integrity, which may have implications for aging-related gut health [134]. Therefore, it could affect brain aging directly, via neuroprotection and mitigation of age-related diseases, and indirectly, by modulating the gut microbiota and decreasing intestinal permeability. However, further pre-clinical and clinical studies are needed to elucidate the exact role of taurine in brain aging and cognition in humans. Additionally, the impact of interindividual variability in taurine levels on cognition merits further investigation. All the key metabolites described above should be validated in clinical studies in order to explore the possibility of their therapeutic use in the mitigation of age-related cognitive decline. The effect of interindividual differences on the regulation of these metabolites should also be taken into account.

## 5. Microbiome and Cognitive Impairment

The gut microbiota plays an important role in maintaining host health by regulating intestinal homeostasis, nutrient metabolism, immunomodulation, and protection against pathogens [135]. It was demonstrated that the gut microbiota interacts with the central nervous system (CNS) in both directions through the complex network, namely, the microbiota-gut-brain (MGB) axis [136]. In turn, the bidirectional MGB communication not only influences brain function and behavior but also allows the CNS to control and regulate gut physiology [113]. The gut-brain axis was intensively studied as it was shown to be involved in the regulation of glia-dependent cognitive functions and considered as a potential target for the treatment of neurological disorders [137]. Communication between the microbiota and the brain may be realized via various mechanisms that include the vagal nerve, enteric nervous system (ENS), immune system, endocrine system, and microbe-derived metabolites [138]. The ENS, also called the “second brain,” is the major component of the MGB axis that mediates gut-brain communication. The ENS is capable of operating independently but also interacts with the CNS, mainly via the vagus nerve, which contains sensory (afferent) and motor (efferent) fibers and relays the information from the gut to the brain and vice versa [44]. The afferent fibers of the vagus nerve receive and transmit the sensory information from the gut to the NTS (nucleus tractus solitarius), and from there, the information may be passed to other brain regions, which are involved in the control of autonomic, cognitive, and behavioral functions [115]. Meanwhile, the gut microbiome synthesizes multiple metabolites that enter the bloodstream and affect the neuronal, endocrine, and immune system pathways. Through these pathways, the gut microbiome affects immune regulation, microglial activation, cytokines, and neuroinflammation [138].

**Figure 3:**
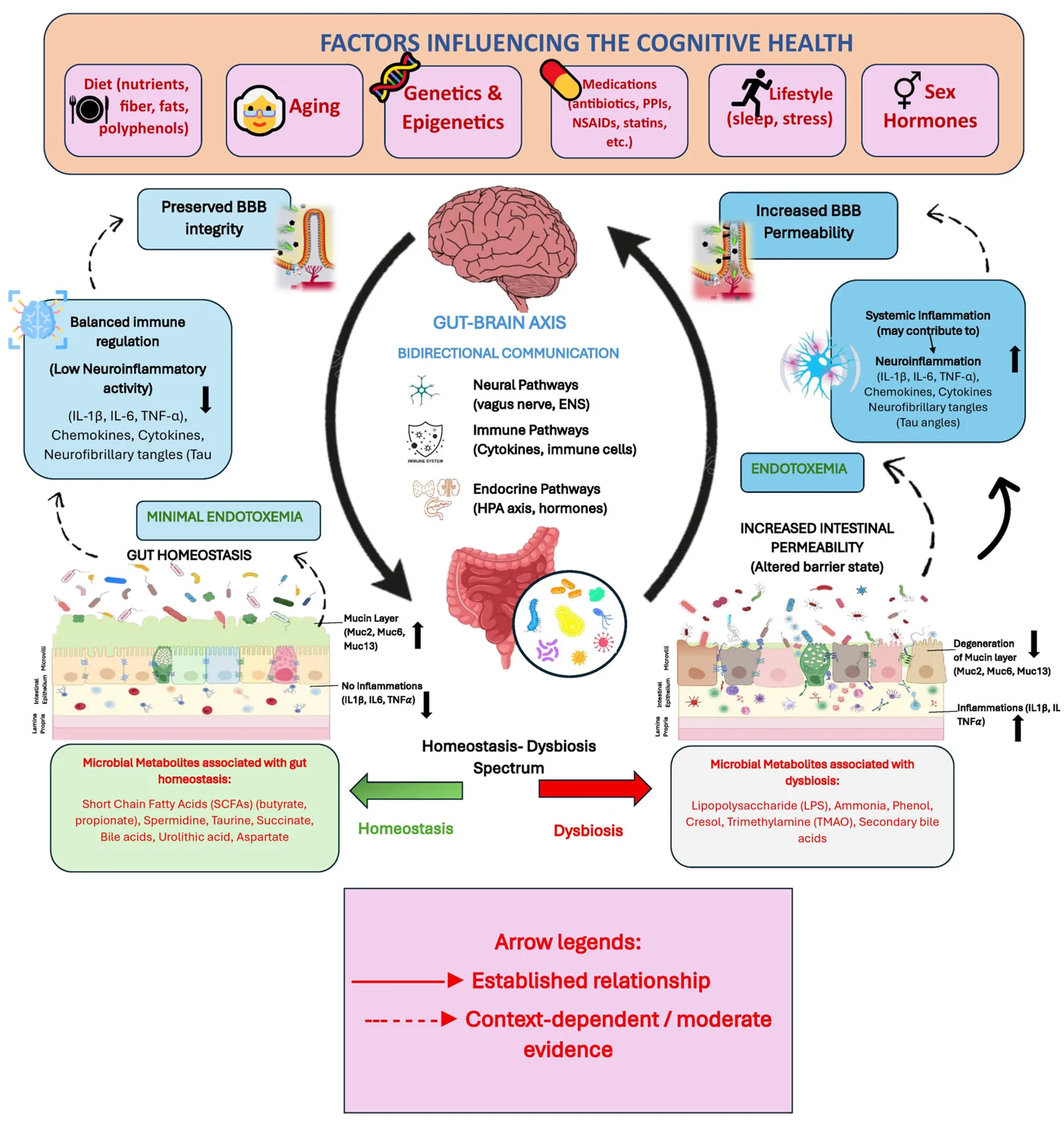
Bidirectional communication between gut and brain in cognitively healthy individuals and states of mild cognitive impairment.

Short-chain fatty acids (SCFAs) are the most extensively researched mediators in the gut-brain axis among the metabolites generated by microorganisms (Figure 3). SCFAs play a role in immune cell function modulation, microglia maturation, gut barrier integrity maintenance, and BBB integrity strengthening [139]. Higher BBB permeability and decreased levels of tight-junction proteins like occludin and claudin-5 have been demonstrated in germ-free mouse models. Following colonization with SCFA-producing bacteria, the defects were partially reversed (Table 2), which strengthens the importance of the gut microbiota in BBB development and maintenance [140]. Although experimental studies have provided mechanistic evidence, such evidence is uncommon in humans.

Aging and a number of neurodegenerative illnesses, including Alzheimer’s disease (AD), have been linked to dysbiosis, which is defined as alterations in the structure and function of the gut microbiome [141]. Dysbiosis is known to alter the microbial communities’ composition, function, and host-microbiota interactions, in addition to simply indicating an increase in the quantity of pathogenic bacteria. Immune homeostatic imbalance, elevated systemic inflammation, and compromised intestinal barrier function have all been connected to this condition and may contribute to the genesis of AD. Studies on animals show that gut dysbiosis is linked to increased levels of pro-inflammatory cytokines, reactive gliosis, amyloid-beta (Aβ) accumulation, and tau phosphorylation, all of which are hallmark pathologies of AD [142]. As for the mechanism of action, gut-derived inflammatory factors can increase systemic endotoxemia, impair blood-brain barrier function, and trigger neuroinflammation; however, much of the data available to date come from animal models [126,143].

Further research has shown that microbial signals regulate the crosstalk between synapse functioning and microglial activation, which affects the development of neurodegenerative diseases and the balance of neurons [144]. Short-chain fatty acids (SCFAs), trimethylamine derivatives, and amino acid metabolites are examples of microbial metabolites that are involved in the neuroendocrine signaling pathways that regulate immune response, neurotransmission, and neuronal functions. By preserving the integrity of the blood–brain barrier, reducing neuroinflammation, and promoting neurogenesis, SCFAs-particularly butyrate-show anti-inflammatory and neuroprotective effects in animal models [145]. However, the conditions in which SCFAs function have a significant impact on their biological effects. The location within the intestine, luminal pH, and metabolite concentration all have different effects on bacterial growth and virulence. For example, whereas higher concentrations in the colon inhibit bacterial growth and suppress the expression of virulence factors such as fliC, ipaH, FimH, and BssS, lower concentrations in the ileum encourage the growth of Escherichia coli [146]. Furthermore, the metabolism of dietary choline, lecithin, and carnitine produces trimethylamine N-oxide (TMAO), which is associated with neurodegeneration and cognitive decline. TMAO may affect neuroinflammatory, metabolic, and tau-related pathways, according to experimental research, but its exact role in AD pathogenesis is still unknown and is probably influenced by host metabolism, dietary factors, and disease stage [145].

Several studies conducted on humans have shown that there is a difference in the composition of gut microbes among individuals suffering from MCI, AD, and cognitively healthy subjects, indicating that the gut microbiome can serve as an important marker for diagnosis and prognosis [147]. However, it should be noted that such conclusions have been made based on observations alone, and so whether microbial changes are the cause or consequence of neurodegeneration is still unclear. Experiments performed on animals have shown the link between gut microbes and processes like amyloid-β aggregation, tau hyperphosphorylation, neuroinflammation, metabolic dysfunction, oxidative stress, and cognitive decline [148].

In summary, current evidence emphasizes the importance of the gut microbiota as an integral part of the microbiota-gut-brain axis that impacts the brain via neurologic, immunologic, endocrinologic, and metabolic pathways. Although experiments support biological feasibility, there is still insufficient direct causal evidence for the association between gut dysbiosis and BBB dysfunction, neuroinflammation, amyloid and tau pathology, and cognitive impairment in humans.

On the left side is represented a healthy gut with intact mucin layers (Muc2, Muc6, and Muc13), decreased intestinal permeability and an anti-inflammatory milieu with decreased levels of IL-1β, IL-6, and TNF-α. Under such conditions, the microbiota produces metabolites with potential neuroprotective properties, such as short-chain fatty acids (SCFAs), spermidine, taurine, bile acids, urolithins, ascorbic acid, and aspartate. There is a lot of experimental and emerging clinical data that support the production and actions of the above-mentioned metabolites. Meanwhile, on the right side is represented gut dysbiosis occurring in cases of mild cognitive impairment, which is characterized by impaired mucin secretion, compromised intestinal barrier functions, and pro-inflammatory cytokine production. Gut dysbiosis can be responsible for the production of harmful microbial metabolites such as lipopolysaccharides (LPSs), ammonia, phenols, cresols, trimethylamine/TMAO, and secondary bile acids. Mechanistically, there are data that indicate endotoxemia, systemic and neuroinflammation, amyloid-β accumulation, tau pathology, and, finally, cognitive impairment, while human observational data indicate the correlation between gut-related factors and cognitive impairment.

## 6. Interconnections Between Aging, Cognitive Decline, and Microbiome

The processes of neuroinflammation, cognitive impairment, endotoxemia, and increased gut permeability are linked to the aging process and the disruption of the balance between the beneficial bacteria and pathobionts. The process of “inflammaging” was introduced to define the chronic low-level inflammation linked to aging and is associated with the presence of recurring tissue injury along with the immune response [21]. Inflammation can be both an influencing factor and a consequence of the alteration in the gut microbiota.

Metabolites from microbes as well as microbial components like double-stranded RNA, lipoproteins, and lipopolysaccharides can trigger innate immunity and contribute to inflammaging-type inflammatory responses, characterized by intestinal inflammation and increased levels of inflammatory cytokines [149]. The mechanism behind this is the ability of LPS to disrupt intestinal epithelial barrier integrity and increase BBB permeability via the TLR4/IRF3 pathway in endothelial cells in blood vessels [150]. Likewise, bacterial components like LPS, amyloid proteins, bacterial lipoproteins, and Bacteroides fragilis toxin have been found to promote microglial activation, impairment of amyloid clearance, BBB dysfunction, and early-stage neuroinflammation related to AD [151,152,153]. In addition, circulating LPS and microbial amyloids may stimulate innate immune receptors like TLRs and RAGE, thus causing more inflammation within the brain and promoting neurodegeneration, especially in areas affected by AD like the hippocampus [154]. Besides the involvement of inflammation, another piece of evidence for the link between gut bacteria, metabolic disorders, and aging of the brain is provided by the complex relationship between these phenomena. Aging-induced changes in the gut microbiota have been linked to insulin resistance (IR) and mitochondrial dysfunction, which might impact brain functionality [155]. Insulin dysfunction in the brain has been shown to increase the risk of neurodegeneration and lead to cognitive deterioration [156]. As follows from these associations, metabolic disorders, including T2D and obesity, have become associated with neurodegenerative disorders, especially AD, in which IR and mitochondrial dysfunction have been recognized as characteristic pathological factors [157]. Moreover, dysbiosis related to IR may impair intestinal permeability and may lead to inflammation, which is associated with the pathophysiology of AD [158]. There is also evidence suggesting that microbial metabolites might be a connection between the gut microbial population and brain functioning. The gut microbiota helps control the generation of neuroactive metabolites, including SCFAs with anti-inflammatory effects and BBB integrity [145]. Furthermore, microbial metabolites might affect neurotransmitter formation in the CNS, thus impacting mood and cognitive functions [159]. These findings have led to increased attention towards the gut-brain axis and development of treatment methods that target this interaction. Probiotics have been suggested as a way of changing the gut microbiota, decreasing inflammation, and slowing down AD development [116].

The interaction between the gut microbiota and cognitive aging is further exemplified by the involvement of the gut microbiota in hippocampal neurogenesis. Aging is characterized by gradual physiologic decline, including genomic instability, mitochondrial dysfunction, and reduced physical activity, and more importantly, it may contribute further to changes in the gut microbiota and cognitive processes [160]. The interrelation between adult hippocampal neurogenesis and the gut microbiome represents another aspect of this interaction. However, this interaction is modulated by numerous environmental and host-specific factors, such as diet, pharmacological intervention, antibiotics, stress, early-life nutrition, genetic background, gender, and geographic location [161]. Although each factor independently affects both gut microbiota and hippocampal functions, several findings demonstrate that the gut microbiota can affect adult hippocampal neurogenesis directly via neural mechanisms including the vagus nerve, or indirectly via microbial metabolites, immune system activation, and hormonal signaling [162]. Moreover, viral-based tracing studies suggest that the gut-associated vagus signals reach the dorsal hippocampus’s glutamatergic neurons and affect hippocampal episodic memory and spatial memory, in part, through alterations in neurogenesis and neurotrophic activity, as represented by DCX and BDNF expression [40,163]. Alterations in the gut microbiota’s composition are additionally associated with hippocampus-mediated learning and memory processes and anxiety [164]. All of these data suggest a multifactorial association between the microbiota, systemic and neuroinflammation, metabolic dysregulation, hippocampal function and aging-associated cognitive decline. However, the degree of evidence is not equal in biological hierarchies. The immune pathways, microbiota-derived metabolites, integrity of the blood–brain barrier, and hippocampal neurogenesis are mechanisms that have been experimentally studied, but there is still insufficient evidence concerning the translation of these mechanisms into cognitive aging and AD in humans. These results, in favor of the role of the microbiota in the maintenance of cognitive health, support the idea about the utility of the modifiable part of the microbiota in the maintenance of cognitive functions and slowing down the progression of the disease [165]. Thus, future research will need to integrate microbiota, biomarkers and cognitive measures in one model at different age stages.

## 7. Interventions

The gut microbiome has been proposed as one possible regulator of healthy aging due to growing evidence that changes in microbial composition and activity could affect cognitive health via multiple routes, such as the immune system, metabolism, endocrinology, and neural mechanisms. As a result, interventions aimed at modulating the gut microbiota (probiotics, prebiotics, synbiotics, postbiotics, diet, ketogenic diets, and FMT) have been actively explored as potential interventions targeting the gut–brain axis [166,167,168,169]. Nevertheless, even considering promising preclinical and early clinical data, such interventions should be considered investigational at this time and cannot yet be used as treatments or preventative strategies for aging-associated cognitive impairment and AD.

**Probiotics:** Probiotics are live microorganisms that improve the host’s health when present in sufficient quantities. Lactobacillus, Bifidobacterium, and Enterococcus are the most frequently studied genera [170,171]. Numerous RCTs found that probiotic administration only slightly improved cognitive function or psychological outcomes, while other studies found little to no impact. Such discrepancy is probably due to variations in the probiotic strain, length of the intervention, dosage, patient demographics, and evaluation of cognitive abilities.

**Prebiotics, synbiotics, and postbiotics**: Prebiotics are non-digestible substances that selectively promote the growth of healthy microbes, while synbiotics represent the combination of probiotics and prebiotics for improving microbial function and survival. Postbiotics include microbial metabolites or microbial cellular constituents that could exert physiological effects independently of the presence of live microorganisms [169,172]. According to experimental studies, such interventions could affect gut microbial metabolism, immune responses, and the synthesis of neuroactive compounds such as SCFAs, GABA, BDNF, and other substances. Clinical studies have also demonstrated that the use of probiotics or synbiotics could reduce inflammatory or metabolic biomarkers, including plasma leptin levels [136,138]. Still, there is little evidence showing the positive effects of such interventions on cognitive functions in elderly people [173]. Next-generation probiotics (NGPs), such as *Akkermansia muciniphila* [174], *Faecalibacterium prausnitzii* [175], *Roseburia intestinalis* [176], and *Bacteroides fragilis* [177,178,179], have been the subject of recent research. This is explained by their capacity to support the integrity of intestinal epithelial cells, the generation of advantageous metabolites, and the control of immune system processes. Additionally, research on prebiotics has progressed from simple prebiotics like inulin, FOS, and GOS to selective prebiotics like *Roseburia, Faecalibacterium*, and *Eubacterium* [138]. Their efficacy and safety in preventing or treating cognitive impairment have not yet been confirmed in human clinical trials, despite encouraging results in experimental studies.

**Dietary interventions:** Diet plays a significant role in determining the composition and function of microbes in the gut. Diets characterized by excessive consumption of fat and sugar have been found to be linked to gut dysbiosis, which predisposes individuals to obesity [4,180,181], type 2 diabetes [131,182], cardiovascular diseases [183,184,185], and cognitive dysfunction [186,187,188]. Meanwhile, diets such as the Mediterranean diet (MedDiet) and the ketogenic diet (KD) have received extensive attention owing to their potential to help individuals age healthily and develop cognitive abilities [114]. Adherence to the MedDiet, which comprises high levels of dietary fiber, polyphenols, and unsaturated fats, has been linked to increased microbial diversity, improved cardiometabolic health, and reduced cognitive decline in humans and primates [184,189]. However, the above benefits are likely achieved through various interconnected processes that include enhanced vascular health, reduced systemic inflammation, metabolic health, and manipulation of the gut microbiome. The KD, which involves high consumption of fat, a moderate amount of protein, and very minimal carbohydrates, has been used to treat individuals suffering from drug-resistant epilepsy [190]. Experimental evidence indicates that the KD can affect the microbial population and possibly the synthesis of metabolites involved in neuronal and immunological signaling, such as SCFAs synthesized from the products of microbial fermentation of the diet. While early results are promising, there is still not enough evidence to suggest using the KD for combating cognitive aging [191].

**Fecal microbiota transplantation:** In order to improve the diversity and functionality of the gut microbiota, FMT involves giving the patient the fecal microbiota of a healthy person. FMT has recently been investigated as a potential therapeutic intervention for age-related disorders and neurodegenerative conditions, despite being a proven strategy for recurrent Clostridium difficile infections [192]. Although it has been noted that some patients with recurrent *C. difficile* infections who receive FMT show improvements in cognitive abilities, it would be incorrect to assume that FMT plays a role in improving cognition in AD patients [193]. Research using animal models of AD also demonstrates that FMT from healthy donors can improve cognitive abilities while lowering neuroinflammation and changing the makeup of the gut microbiota [194]. Metabolites generated by the gut microbiota, such as the byproducts of the tryptophan–kynurenine signaling pathway involved in neurotransmission and neuroinflammation, may facilitate communication between the gut and the brain [195,196]. The role of the gut microbiome in the development of AD has also been confirmed by other experiments with germ-free and antibiotic-treated mice [197]. It has also been discovered that microbiota transplanted from genetically modified long-lived Ames dwarf mice increase beneficial microbes and influence host gene expression linked to longevity and improved hepatic function [198]. Clinical trials must be carried out to determine the effectiveness of FMT treatment for preventing cognitive decline in humans, even though these results provide compelling evidence for the mechanism of action.

Together, these findings imply that manipulation of the gut microbiota through specific interventions can affect biological pathways related to brain aging, such as immune regulation, production of metabolites by the microbiota, intestinal integrity, and neuroinflammation, among others. However, much of this information comes from either animal studies or small and heterogeneous clinical trials. Longitudinal randomized controlled trials are required to confirm whether manipulation of the gut microbiota would result in clinically relevant benefits on cognition and cognitive decline associated with aging. In conclusion, progress in sequencing and metagenomics has greatly increased knowledge about the composition of the gut microbiota and its implications for healthy aging. While microbiota-targeting interventions seem very promising, they have yet to be proven safe and effective through clinical and mechanistic studies.

## 8. Limitations and Future Direction

Despite the significant progress in studying microbiota-brain connectivity, numerous challenges are associated with the use of microbiome signatures as clinical predictors. First of all, most of the studies exploring the relationship between microbiome alterations and cognitive impairments are cross-sectional. In addition, many researchers used small sample sizes, making the results difficult to generalize to the broader population. Moreover, most of these studies have a limited set of confounding variables, which decreases the accuracy of findings. It is hard to say whether the identified patterns reflect the cause-and-effect relationship between microbiome changes and cognitive decline or whether they are only secondary to other variables. Apart from that, there may be variations in participants’ characteristics, geographical location, dietary habits, treatment, and other factors. Another challenge is the lack of consistency in the study and pre-processing design, including the selection of the sequencing platform, sample preparation, and processing methods. As a result, only a few microbes or metabolites identified as promising biomarkers of cognitive function have undergone the required validation. Therefore, to ensure the reproducibility and reliability of findings across different populations and settings, future microbiome studies should employ standardized methods. Moreover, to identify causal links, longitudinal studies that aim to assess the impact of microbiome alterations on cognitive functions and overall brain health should be conducted. Such trials ought to include diverse cohorts of participants as well as processing numerous biological, cognitive, and neuroimaging endpoints.

Future research on the gut microbiota and its impact on the human brain and aging requires a multi-omics approach, including an integrated analysis of meta-genomics, meta-transcriptomics, metabolomics, proteomics, epigenomics, and host transcriptomics to identify the mechanistic links between the gut microbiome and the key domains of aging. The combined application of microbiome data and the state-of-the-art technologies of artificial intelligence and machine learning can enable the identification of biomarker modules associated with the risk of cognitive decline. Mechanistic interventional human studies, including randomized controlled trials, are also needed to evaluate the potential of dietary, probiotic, prebiotic, postbiotic, microbiota-derived metabolite-based and fecal microbiota transplant approaches to modulate the gut microbiome and its impact on brain aging. Biomarker modules derived from microbiome, transcriptomic, metabolomic and other ‘omics’ data, including traditional biomarkers of aging, cognition, genetics, and neuroimaging can serve as a framework for precision microbiome medicine to identify individuals at risk and initiate individualized dietary, pharmacologic or microbiome-based interventions to maintain cognitive health and prevent age-related cognitive decline.

## 9. Conclusions

The concept of the gut-brain-microbiota axis is the biological entity through which the function of the intestinal wall, the metabolic processes of microbes, immune responses, and neuroendocrine pathways can affect cognitive aging and MCI. The research evidence presented in this article indicates that there is a biologically plausible link between the presence of dysbiosis, changes in metabolites produced by microbes, inflammation, the impaired function of the blood–brain barrier, and neurodegeneration. Yet, the quality of evidence varies greatly from experiments to clinical observations.

Several testable hypotheses can be formulated based on the existing body of evidence. Hypothesis one is that subjects suffering from MCI would show predictable shifts in pre-specified groups of gut microbes and metabolites in comparison with their cognitively healthy age counterparts, regardless of important dietary, medication, and metabolic variables. Hypothesis two is that a multimodal biomarker system that includes gut microbial profiling, microbial metabolites, inflammatory factors, and neurological biomarkers will have better discriminatory power for MCI than any single microbial or metabolite biomarker. Hypothesis three is that longitudinal modifications in the preselected microbial and metabolite markers would predict future changes in cognitive functions and other established neurodegenerative biomarkers. Hypothesis four is that restoration of gut barrier function or targeted interventions of microbial metabolic pathways will lead to predictable changes in systemic inflammation and neurodegeneration biomarkers, thus establishing a causal relationship between the two.

Taking into consideration all of the evidence collected above, one can suggest a preliminary framework for research-to-clinical application of this information. On the first level, people who have subjective cognitive complaints or those who are considered to be at risk of cognitive decline can be assessed using clinical and cognitive testing as well as the testing of their metabolic and vascular risk factors. On the second level, people who match the criteria for higher risk of cognitive decline will be assessed using established biomarkers, when needed, along with research-level testing of their gut microbiome composition and metabolite levels, intestinal inflammation/leakage markers, and biological age indicators. On the third level, these variables can be combined into a multimodal risk model, which will allow assessing the cognitive risk status of an individual in a standardized way. Critically, the proposed algorithm at present is merely a research tool and not a clinically validated decision tool. Microbiome-based biomarkers do not yet replace well-established neurological, radiological, and cognitive tests, and no single microbe or metabolite can be considered a clinically validated biomarker without proof of sensitivity, specificity, AUC, reproducibility, and external validation in humans.

Consequently, future research must aim to implement prospective, longitudinal study designs; use rigorous microbiome and metabolomics methodologies; control for dietary and medication-related confounding; use pre-specified criteria for diagnosis; and conduct validation in independent cohorts. A special focus must be placed on determining the incremental predictive power of microbiome-derived biomarkers above well-established risk factors. In the end, the clinical usefulness of the microbiota-gut-brain axis model will depend not only on the identification of biomarkers associated with cognitive decline but also on validation of the clinical use of these biomarkers in terms of improving predictions or treatment decisions in patients.

## Figures and Tables

**Table 1:** Human evidence on aging, cognitive impairment, and microbiome-related biomarkers and interventions.

Study Model and Clinical Condition	Interventions	Biomarkers	Research Outcomes	References
Observational biomarker study: elderly with suspected Alzheimer’s disease	Diagnostic evaluation	CSF Aβ42, p-tau181/217/231, and NfL	Accurately detects early AD pathology via CSF and imaging	[35,36,37]
Longitudinal observational study: older adults with MCI	None (biomarker panel study)	Plasma Aβ42/40, p-tau, NfL, IL-6, CRP, and TNF-α	Predicts conversion to AD; correlates with brain atrophy and synapse loss	[38]
Intervention study: aging individuals with mild cognitive decline	Probiotics, prebiotics, and synbiotics	IL-6, TNF-α, CRP, p16INK4a, Tau, and SNAP-25	Reported associations with improved cognitive outcomes and reduced inflammatory markers	[8,39,40]
Metabolic intervention: patients with neurodegenerative symptoms	Spermidine, taurine, and metformin	CRP, IL-6, TNF-α, and SASP	Reduces senescence, restores mitochondrial integrity, and supports cognition	[41,42]
Genetic study: familial AD risk groups	Genetic profiling	APOE ε4, TREM2, PSEN1, and PSEN2	Identifies genetic susceptibility	[43]
Biomarker study: early AD cases	None	Synaptophysin, SNAP-25, and GAP-43	Detects synaptic degeneration	[7,44]
Human-tissue-based study: aging human brain tissue samples	None	p16INK4a, telomeres, and SASP	Confirms cellular aging	[8,9]
Biomarker study: serum of aging adults	None	IGF-1, mTOR, ROS, and SOD	Shows mitochondrial decline	[45,46]
Dietary/microbiome intervention: geriatrics under diet/microbiome therapy	Probiotics, inulin, and Mediterranean/ketogenic diets	SCFAs, TMAO, taurine, and spermidine	Reported changes in microbial metabolites have demonstrated significant cognitive health improvement	[40,47]
Longevity cohort study: long-lived human cohorts	Fiber-rich diets and fermented foods	Microbiota diversity, SCFAs, and IL-1β	Longevity linked to preserved microbiota	[48]

**Table 2:** Preclinical evidence from in vitro, in vivo, and experimental models linking aging, cognition, and the gut microbiome.

Evidence Type	Study Model	Intervention/Exposure	Biomarkers/Measures	Research Outcomes	References
Preclinical-in vitro	Neuronal cell model	Fenchol (50–100 μM)	N/A	Reduced Aβ-induced neurotoxicity and cellular senescence	[42]
Preclinical-in vivo	*C. elegans* model	Fenchol	Aβ accumulation	Reduced Aβ accumulation	[42]
Preclinical-animal	Germ-free mouse model	SCFA-producing bacteria	Occludin and claudin-5	Restoration of blood–brain barrier (BBB) integrity	[108,113]
Preclinical-animal	APP/PS1 mouse model of AD	Fecal microbiota transplantation (FMT)	Aβ plaques and microglial activation	Reduced amyloid pathology and improved cognitive performance	[114]
Preclinical-animal	Middle-aged mouse model	Taurine	p16^INK4a and mitochondrial DNA damage	Reduced markers of cellular aging	[42]
Preclinical-in-vitro	Senescent fibroblast model	Metformin	SASP and p16^INK4a	Reduced pro-inflammatory secretory activity	[26,92]
Preclinical-animal	Mouse model of neuroinflammation	Dietary fiber and FMT	TMAO, IL-1β, and SCFAs	Improved BBB integrity and synaptic plasticity	[90,115]
Preclinical-in-vitro	Cellular senescence model	Oxidative stress assays	p16^INK4a and SASP	Increased/chronic inflammatory phenotype	[8,31]
Preclinical-in- vitro/experimental	Mitochondrial models	ROS-related experimental assays	ROS, mtDNA, and SOD	Mitochondrial dysfunction associated with neurodegenerative processes	[50,51]
Preclinical-epigenetic/in silico	Epigenetic aging models	DNA methylation clocks	CpG methylation	Estimation/prediction of biological age	[28]
Preclinical-microbial screening	Microbial screening models	*A. muciniphila* and *F. prausnitzii*	SCFAs	Identification of potentially beneficial bacterial strains and metabolites	[40]
Review of preclinical evidence	AD mouse models	FMT	SCFAs and Aβ	Evidence for reduced neuroinflammation in experimental models	[23,114]
Preclinical-animal	Antibiotic-treated mouse model	Antibiotic exposure	Gut microbial diversity	Altered microbial diversity and impaired learning	[116]
Preclinical-in vivo	*C. elegans* aging model	Spermidine	Longevity-related measures	Extended lifespan	[93]

Note: The data from cell cultures, *C. elegans*, and animals are considered preclinical data and cannot be regarded as data for the effects in humans. Human observational and clinical studies are provided in a separate section, and the terms “association” and “effect” are used depending on the case. Abbreviations: AD, Alzheimer’s disease; Aβ, amyloid-β; BBB, blood–brain barrier; FMT, fecal microbiota transplantation; ROS, reactive oxygen species; SASP, senescence-associated secretory phenotype; SCFAs, short-chain fatty acids; TMAO, trimethylamine N-oxide.

## Data Availability

No new data were created or analyzed in this study. Data sharing is not applicable to this article.

## Abbreviations

AD	Alzheimer’s disease
Aβ plaques	Amyloid beta plaques
GBA	Gut–brain axis
BBB	Blood–brain barrier
FMT	Fecal microbiota transplantation
MCI	Mild cognitive impairment
CSF	Cerebrospinal fluid
APOE	Apolipoprotein E
TREM 2	Triggering receptor expressed on myeloid cells 2
SNAP 25	Synaptosome-associated protein 25
SASP	Senescence-associated secretory phenotype
CRP	C-reactive protein
IL 6	Interleukin 6
TNFα	Tumor necrosis factor alpha
IGF 1	Insulin-like growth factor 1
mTOR	Mammalian target of rapamycin
F/B	Firmicutes/Bacteroidetes
SCFAs	Short-chain fatty acids
TMAO	Trimethylamine N-oxide
CNS	Central nervous system
HPA	Hypothalamic–pituitary–adrenal
MedDi	Mediterranean diet
KD	Ketogenic diet
CVD	Cardiovascular disease
ROS	Reactive oxygen species
Esasp	Exosomal SASP
sSASP	Soluble SASP
DNAm	DNA methylation
SOD	Superoxide dismutase
CT	Computed tomography
MRI	Magnetic resonance imaging
PET	Positron emission tomography
WMHs	White matter hyperintensities
SPARE-BA	Spatial pattern of atrophy for recognition of brain aging
NFTs	Neurofibrillary tangles
p-tau	Phosphorylated tau
NFP	Neurofilament protein
NfL	Neurofilament light protein
ALS	Amyotrophic lateral sclerosis
MTHFR	Methylenetetrahydrofolate reductase
PPARG	Peroxisome proliferator-activated receptor gamma
GCKR	Glucokinase regulatory protein
GAP	Growth-associated protein
MMSE	Mental state examination
MoCA	Montreal Cognitive Assessment
AMTS	Abbreviated mental test score
ADAS-Cog	AD assessment scale-cognitive
GPCOG	General practitioner assessment of cognition
NCD	Neurocognitive disorder
VN	Vagus nerve
ENS	Enteric nervous system
NTS	Nucleus tractus solitarius
GABA	Gamma-aminobutyric acid
NGPs	Next-generation probiotics
